# Supplementary material for: The ‘Shape-Shifter’ Peptide from the Disulphide Isomerase PmScsC Shows Context-Dependent Conformational Preferences
Source: Biomolecules. 2021 Apr 26;11(5):642. doi: 10.3390/biom11050642 (PMC8146718; doi:10.3390/biom11050642)
Supplement: Supplementary file 1 [file biomolecules-11-00642-s001.zip › Supplementary/SupplementaryMaterial_TableS1.pdf]

Table S1. The results of clustering the 40000 conformers taken from the four 100 ns MD simulations of the linker peptide. The number of conformers of each simulation trajectory present in each of the seven main clusters identified, the hydrogen bonds present in the conformers of each cluster (populations > 70%) and present in the structure that is the centre of each cluster are listed (excluding those from the acetyl or amide groups at the N and C- termini). The conformational clustering was performed using the algorithm of Daura et al. [24] and the backbone N, CA and C atoms of residues 3-11. The atom positional RMSD cutoff used to determine the structures belonging to a single cluster was 0.15 nm.

C1uster number	Starting conformation				Hydrogen bonds with population > 70% in cluster	Hydrogen bonds in cluster centre structure
	5id4	5idr_A	5idr_B	4xvw		
1	278	0	0	8343	40 NH – 49 O, 42 NH – 39 O, 47 NH – 38 O	38 HZ2 – 42 OE2, 39 HZ1 – 49 OE1, 40 NH – 49 O, 42 NH – 39 O, 45 NH – 42 O, 47 NH – 45 O
2	1	7775	3	0	45 NH – 41 O	44 NH – 41 OD1, 44 HE22 – 41 OD2, 45 NH – 41 O, 48 NH – 45 O, 50 NH – 48 O
3	1386	0	3853	0	46 NH – 42 O, 47 NH – 43 O	41 NH – 39 O, 42 NH – 38 O, 44 NH – 42 OE1, 44 NH – 42 OE2, 44 HE22 – 42 OE1, 45 NH – 42 OE2, 46 NH – 42 O, 47 NH – 43 O
4	2039	281	2218	0	44 NH – 40 O, 45 NH – 41 O, 46 NH – 42 O, 47 NH – 43 O,	42 NH – 38 O, 43 NH – 39 O, 44 NH – 40 O, 45 NH – 41 O, 46 NH – 42 O, 47 NH – 43 O,

						48 NH – 46 O
5	643	0	208	482	43 NH – 39 O, 44 NH – 40 O,	39 NH – 42 OE2, 39 HZ1 – 41 OD1, 43 NH – 39 O, 44 NH – 40 O, 45 NH – 41 O, 48 HE – 46 OE1
6	1303	12	7	0	44 NH – 40 O, 46 NH – 41 O	38 HZ3 – 41 OD1, 40 NH – 44 OE1, 41 NH – 41 OD2, 44 NH – 40 O, 45 NH – 40 O, 46 NH – 42 O, 46 HE22 – 41 O
7	7	1263	0	0	46 NH – 41 O	43 NH – 41 OD2, 44 NH – 41 OD2, 46 NH – 41 O, 46 HE22 – 40 O, 48 HE – 43 O, 48 HH22 – 43 O
