# Supplementary material for: The ‘Shape-Shifter’ Peptide from the Disulphide Isomerase PmScsC Shows Context-Dependent Conformational Preferences
Source: Biomolecules. 2021 Apr 26;11(5):642. doi: 10.3390/biom11050642 (PMC8146718; doi:10.3390/biom11050642)

Figure S3. Far-UV circular dichroism (CD) spectra collected for the shape-shifter peptide as a function of trifluoroethanol (TFE) concentration. An increase in helical content as the TFE concentration is increased from 0% to 50% is evident from the negative peaks that emerge at 208 and 222 nm. Far-UV CD measurements in the 190-260 nm wavelength range were carried out at 15 °C using a Jasco J-815 CD spectrophotometer. The peptide concentration was ~50μM and a cuvette of 0.1 cm path length was used. Samples contained v/v mixtures of 0.01M Na_2HPO_4 buffer and TFE at pH 7. The CD spectra were collected with 0.5 nm resolution, with a scan speed of 50 nm/min, and are the average of 4 measurements.

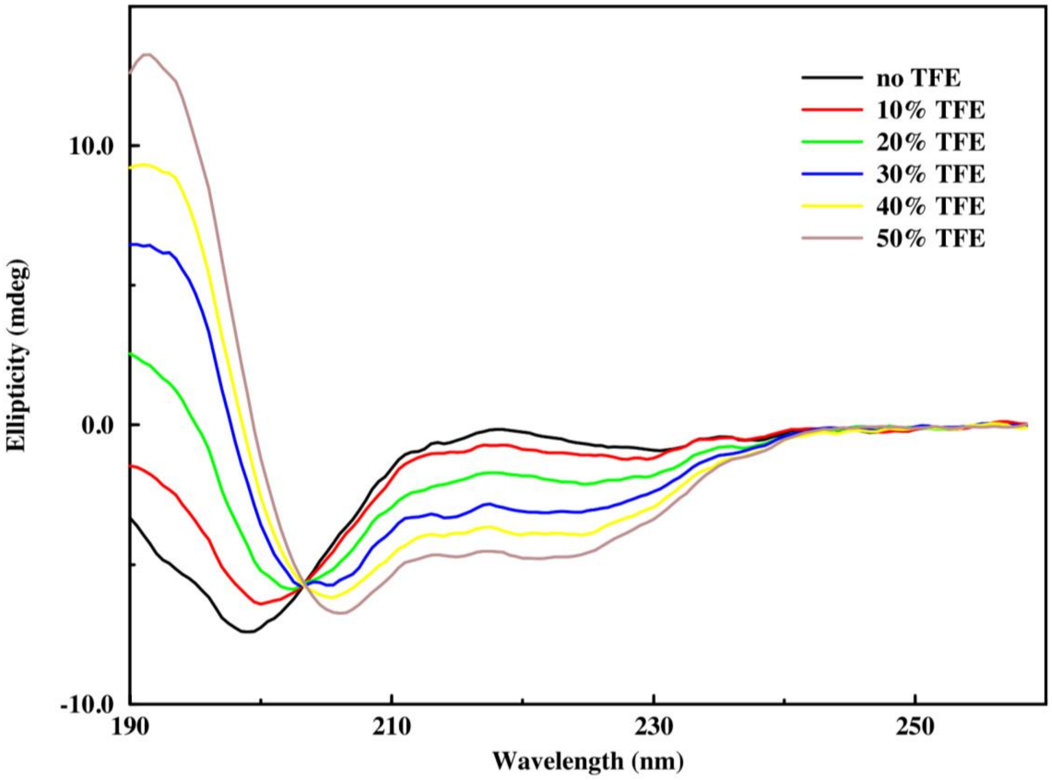

Supplement: Supplementary file 1 [file biomolecules-11-00642-s001.zip › Supplementary/SupplementaryMaterial_FigureS3.pdf]
