# Supplementary material for: The ‘Shape-Shifter’ Peptide from the Disulphide Isomerase PmScsC Shows Context-Dependent Conformational Preferences
Source: Biomolecules. 2021 Apr 26;11(5):642. doi: 10.3390/biom11050642 (PMC8146718; doi:10.3390/biom11050642)

Figure S2. Overlay of the natural abundance ^{1}HN-^{15}N HSQC spectra of the shape-shifter peptide in H_2O (black) and in 50% TFE (red) at pH 7 collected at 600 and 500 MHz, respectively. Peaks are labelled with their sequence-specific assignments.

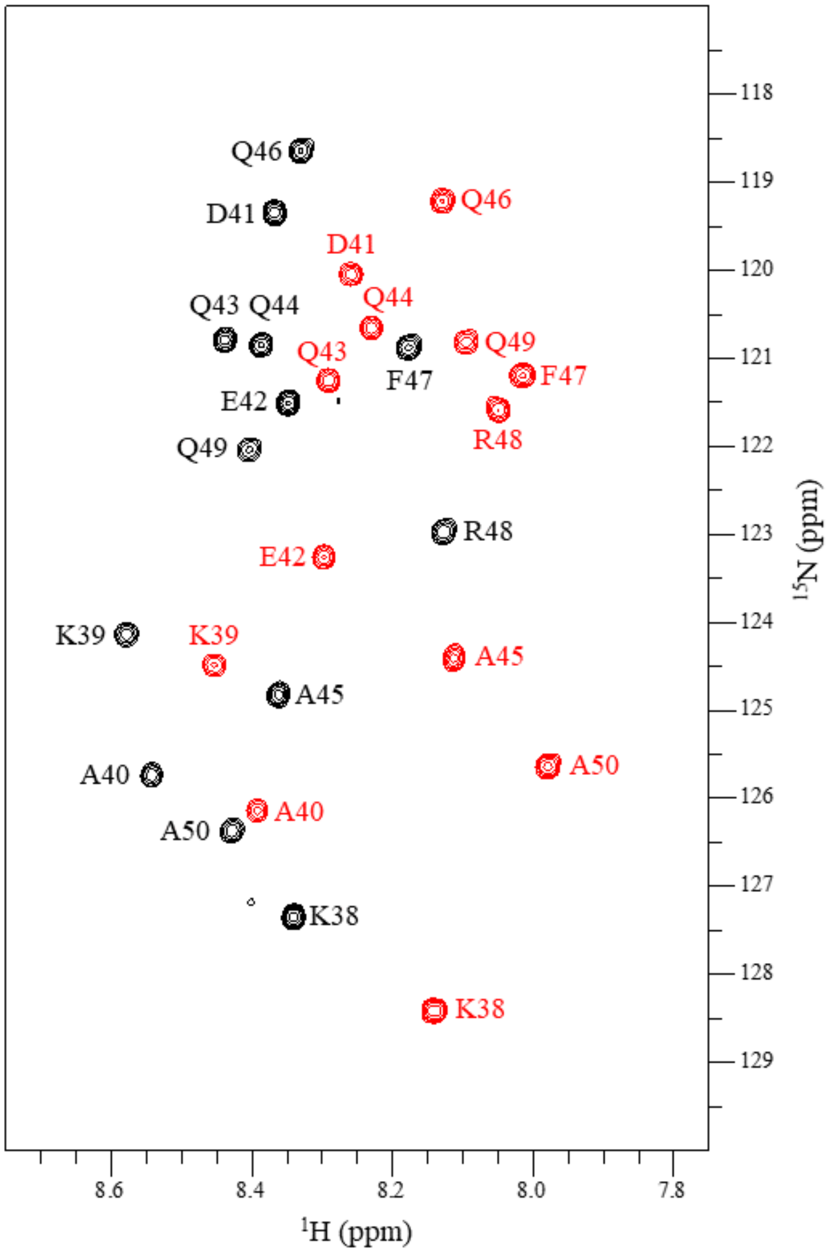

Supplement: Supplementary file 1 [file biomolecules-11-00642-s001.zip › Supplementary/SupplementaryMaterial_FigureS2.pdf]
