# Supplementary material for: The ‘Shape-Shifter’ Peptide from the Disulphide Isomerase PmScsC Shows Context-Dependent Conformational Preferences
Source: Biomolecules. 2021 Apr 26;11(5):642. doi: 10.3390/biom11050642 (PMC8146718; doi:10.3390/biom11050642)

Figure S1. Overlay of the $^{1}H\alpha$-$^{13}C\alpha$ region of the natural abundance ^{1}H-^{13}C HSQC spectra of the shape-shifter peptide in H_2O (black) and in 50% TFE (red) at pH 7 collected at 600 and 500 MHz, respectively. Peaks are labelled with their sequence-specific assignment. The increase in helical propensity in 50% TFE can be seen clearly by the upfield shift of $^{1}H\alpha$ and the downfield shift of $^{13}C\alpha$ in TFE relative to H_2O buffer.

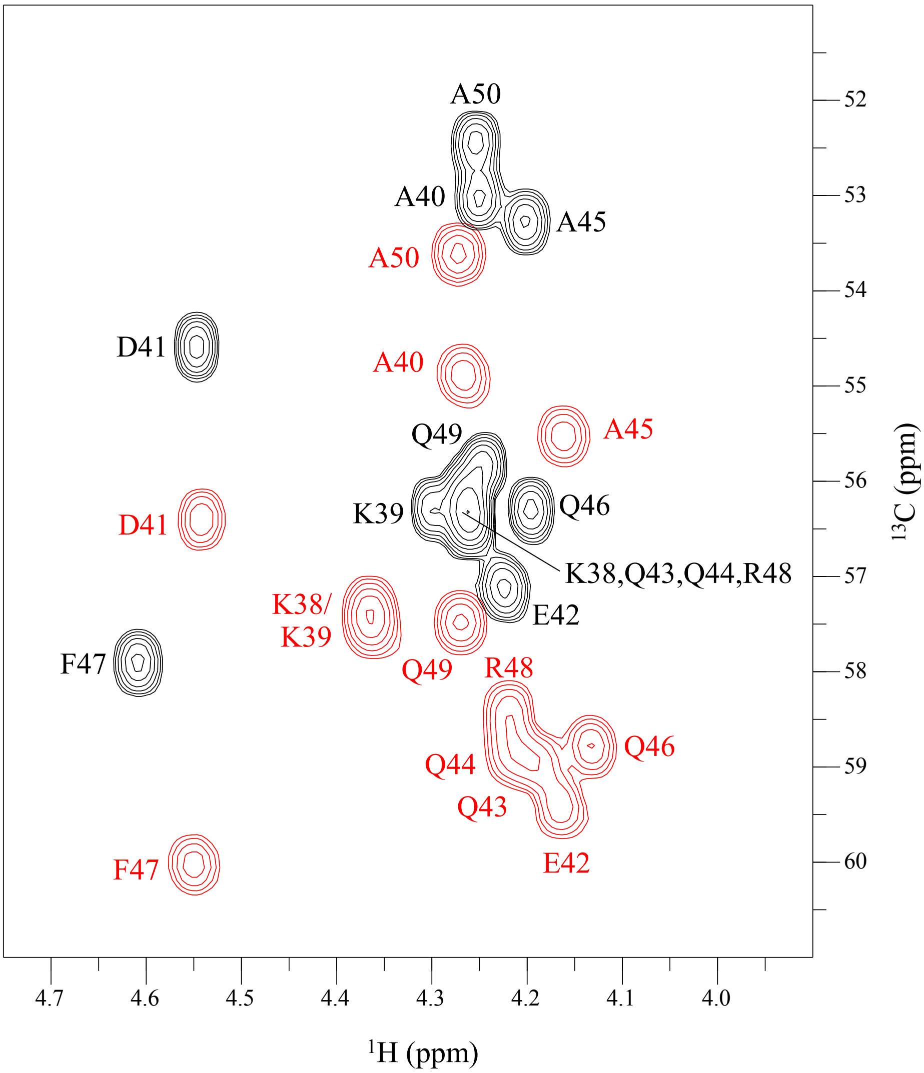

Supplement: Supplementary file 1 [file biomolecules-11-00642-s001.zip › Supplementary/SupplementaryMaterial_FigureS1.pdf]
